# The Johns Hopkins Hospital of Baltimore City

**Published:** 1877-08

**Authors:** 


### EDITORIAL, ETC.

---

*The Johns Hopkins Hospital.*—Baltimore City will soon have the grandest Hospital in the country.

"On June 23d Mr. Francis T. King, president of the Board of Trustees of Johns Hopkins Hospital, turned up the first sod to begin the building of the hospital which is to occupy over a block of ground in the eastern section of the city. The proceeding was entirely informal and was done at the instance of the workmen and in their presence. Prayer was offered for God's blessing, and Mr. King made a few remarks in regard to the plan and the object of the hospital. He then turned the sod at the centre of the administration or main building, which will occupy the most prominent location on the grounds, facing on Broadway, midway, at McElderry Street. It is to be of compressed brick, with 290 feet front, on Broadway and 90 feet regular depth. Projections of bay windows reaching to the roof will increase the depth at the ends and centre to 125 feet. It will be three stories high and trimmed with Cheat river stone like the Safe Deposit Company building on South Street. Two structures of the same material each 130 by 90 feet, will also face on Broadway both slightly in the rear of the centre building, one north and the other south of it. The one on the north at the Monument Street corner, will be known as the female pay ward, and that on the south, at Jefferson Street, as the male pay ward, both for the reception of patients able to pay for their accommodations. In the rear of the female pay ward, fronting on Monument Street, will stand the kitchen, to be of brick, 75 feet square. The nurses' home and building for the training and instruction of nurses and attendants will occupy a corresponding position facing on Jefferson Street, in the rear of the male pay ward. This building will be 100 feet square.

The officers' quarters, dining rooms, &c., will occupy a building 50 by 80 feet, to the east and north of the administration building. The house of the resident physician will stand east of the kitchen and female pay ward on Monument Street, and will be 50 by 75 feet in dimensions. East of this is to be located the operating theatre, as it will be known, an extensive structure, with one high story above the basement, 200 by 75 feet. The last building in this row on Monument Street will be the dead-house, at the corner of Wolf Street. Beginning at the rear of the central administration building a grand avenue, 170 feet wide, is to lead to Wolf Street, through the centre of the grounds. On either side of this avenue, occupying very nearly the centre of the grounds, will stand five common wards for the reception of general patients. Each of these will be 466 by 160 feet, and will stand 60 feet apart. Two octagonal bath-houses will stand between the administration building and the pay wards. The ground plan thus briefly stated provides for two rows of buildings on the north of the grand avenue and one row south, as the square between McElderry Street and Monument Street is longer than that between McElderry Street and Jefferson Street. This will leave ninety-two feet space on Jefferson Street from the building line. The plan for the use of this ground and that not occupied by other buildings in lawns, terraces, beautiful gardens, ponds, fountains, &c., is very extensive and elaborate. On the Wolf Street side will remain a front of 113 feet, which will probably be used for ambulance houses, &c. All the buildings will be connected with the administration hall by a wide iron corridor running from one structure to another. The entire grounds will be surrounded by an open iron railway four feet high, through which and over which a view of the premises may be gained.

The excavating for the foundation, begun by Mr. King, will be pushed rapidly forward. The seventeen buildings which stood on the southwest corner have been sold and the material almost entirely removed. The full survey of the grounds has been made and foundation points and lines for the several buildings marked with stakes. The grand avenue is being opened and the preparatory work is being pushed with vigor. The work is in charge of Dr. H. Billings, medical director; John R. Niernsee, architect, and John E. Marshall, superintendent."

